# Comparison of efficacy of three commonly used alpha-blockers as medical expulsive therapy for distal ureter stones: A systematic review and network meta-analysis

**DOI:** 10.1590/S1677-5538.IBJU.2020.0548

**Published:** 2021-02-20

**Authors:** Gopal Sharma, Tarun Pareek, Pawan Kaundal, Shantanu Tyagi, Saket Singh, Thummala Yashaswi, Sudheer Kumar Devan, Aditya Prakash Sharma

**Affiliations:** 1 PGIMER Advanced Urology Centre Department of Urology Chandigarh India Department of Urology, Advanced Urology Centre, Level II, B-Block, PGIMER, Chandigarh, India

**Keywords:** Therapeutics, Adrenergic alpha-Antagonists, Calculi, Meta-Analysis [Publication Type]

## Abstract

**Introduction:**

The efficacy of alpha-blockers as medical expulsive therapy (MET) is well established. However, it is not known which of the three most commonly used alpha-blockers (tamsulosin, alfuzosin and silodosin) is the most efficacious. With this study we aimed to assess the efficacy of the three commonly used alpha-blockers as MET for distal ureter stones.

**Materials and Methods:**

For this review, we searched multiple databases such as PubMed/Medline, Scopus, Embase, OviD SP, CINAHL, and web of science to identify all the relevant randomized studies comparing the efficacy of tamsulosin, alfuzosin, and silodosin. Preferred reporting items for systematic reviews for network meta-analysis (PRISMA-NMA) were followed while conducting this review and the study protocol was registered with PROSPERO (CRD42020175706).

**Results:**

In this review, 31 studies with 7077 patients were included. Compared to placebo all the treatment groups were more effective for both stone expulsion rate (SER) and stone expulsion time (SET). For both SER and SET, silodosin had the highest SUCRA (94.8 and 90.4) values followed by alfuzosin (58.8 and 64.9) and tamsulosin (46.2 and 44.5). The incidence of postural hypotension was similar with all the drugs, whereas, the incidence of retrograde ejaculation was significantly higher for silodosin. Overall confidence for each comparison group in this review ranged from “very low” to “moderate” according to the CINeMA approach.

**Conclusion:**

Among the three commonly used alpha-blockers silodosin is the most efficacious drug as MET for lower ureter stones followed by alfuzosin and tamsulosin.

## INTRODUCTION

The worldwide prevalence of stone disease is gradually increasing in recent times and presently it is estimated to be 5-10% (1). Ureteric stones, as a subset of urinary stones, demand prompt diagnosis and treatment due to their propensity to cause back pressure changes leading to obstructive uropathy, if not relieved timely.

Spontaneous expulsion of ureteric stones depends on diverse factors, of which stone size and location remain the most pertinent predictors for stone passage (2). Failure of spontaneous passage of ureteric stones necessitates intervention. The current treatment modalities for ureteric stones include conservative measures such as medical expulsive therapy (MET) and extracorporeal shockwave lithotripsy (ESWL) and surgical interventions including endoscopic, open surgery, laparoscopic surgery, and robot-assisted surgery. As per the latest American and European guidelines, MET remains a feasible management option for ureteric stones less than 10mm, given its non-invasive and comparatively inexpensive features (3-5).

Multiple drugs such as α-blockers (tamsulosin, silodosin, alfuzosin, and naftopidil) (6-8), calcium channel blockers (CCB) (nifedipine) (9) and phosphodiesterase inhibitors (PDEI) (sildenafil and tadalafil) (10, 11) have been found to be effective in facilitating the expulsion of ureteric stones compared to general measures such as the use of non-steroidal anti-inflammatory drugs (NSAIDS), hydration, antispasmodics, diuretics, and placebo. Most of the evidence has been published with alpha-blockers (6, 7). However, among various available alpha-blockers the comparative efficacy and safety has been a matter of debate. Few studies have compared different alpha-blockers to each other (12) and there is a paucity of studies evaluating the relative efficacy of individual alpha-blockers specifically for “distal” ureteric stones, as MET has been reported to be most efficacious for this subgroup compared to proximal ureteric location (4). Thus, with this study we aimed to compare the relative efficacy of three commonly used alpha-blockers (alfusozin, silodosin and tamsulosin) as MET for distal ureteric stone.

## MATERIALS AND METHODS

This systematic review and network meta-analysis were performed with a frequentist approach. A pre-specified study protocol was registered with PROSPERO (CRD42020175706) and standard Preferred Reporting Items for Systematic reviews and Meta-analysis (PRISMA) guidelines for conducting network meta-analysis (NMA) were followed (13).

### Literature search

Systematic literature search for various electronic databases such as PubMed/Medline, Scopus, Embase, OviD SP, CINAHL and web of science was conducted by two study authors independently (GS & ST). A literature search was conducted from the time of inception of these databases until March 2020. Literature search was limited to English only. The search string used for literature search was based on Patient, Intervention, Control and Outcome (PICO) guidelines. The following keywords and strategy were used: Patient: Lower ureteric stone OR Lower ureteric calculi OR Distal ureteric stone OR Distal ureteric calculi. Intervention: alfuzosin OR silodosin OR tamsulosin. Control: No treatment. Outcome: Stone expulsion OR medical expulsive therapy.

The search results thus obtained from various databases were transferred on to a citation manager and additional articles were also sought from various review articles on same topic and hand searches of references selected for full-text review were also undertaken.

### Study eligibility criteria

Following a comprehensive literature search, initial title and abstract screening were conducted by two authors independently (GS & ST) to screen the articles for possible inclusion into the study based on the below mentioned exclusion and inclusion criteria.

Inclusion criteria:

- Randomized studies containing data on the number of stone expulsion or time to stone expulsion in adult patients with lower ureteric stones with the use of any of the three alpha- blockers being studied. Comparison could be against control group or with each other.

Exclusion criteria:

1. Non-randomized studies
2. Case reports, editorials, letters, reviews and conference abstracts
3. Not containing data on above mentioned drugs i.e. silodosin, alfuzosin ortamsulosin.
4. Studies in the pediatric age group (age <18 years).
5. Not containing data on the stone expulsion rate (SER) or stone expulsion time (SET) at the completion of study.

Studies were then selected for full-text review and those satisfying the inclusion and exclusion criteria were included in review. In case of disagreement between two study authors, arbitration with the other authors was done.

### Data extraction

Two study authors independently (GS & ST) extracted data from the studies on a pre-determined format including following variables such as first author, year, type of study, country, type of treatment, duration of treatment, baseline comparability according to age, sex, stone size, SER and SET. The discrepancy of data was resolved after arbitration with other study authors.

### Outcome

The primary outcome studied was SER at the end of study period in the treatment and the control groups. We also provided data for ranking of the three alpha-blockers on their efficacy for the expulsion of distal ureteric stones in terms of SER Data on SET and complications such as postural hypotension and premature ejaculation was also analyzed in this study and various alpha-blockers were ranked accordingly (secondary outcome).

### Statistical analysis and certainty of evidence

This network meta-analysis (NMA) was performed using frequentist approach that determines the probability of an event to occur if the same process is repeated multiple times (14, 15). This NMA was designed to compare three treatment groups (alfuzosin, silodosin and tamsulosin) for the primary and secondary outcomes. This NMA is aimed to combine both direct and indirect evidence into a single effect size for the two comparisons i.e. stone expulsion rate and time to stone expulsion. Relative rankings of various alpha-blockers i.e. alfuzosin, silodosin and tamsulosin were estimated for both the outcomes using the distribution of ranking probabilities and surface under the cumulative ranking area curves (SUCRA). For publication, bias visual interpretation of comparison adjusted forest plots was done. All the statistical analysis was performed using Stata (version 16; StataCorp, College Station, TX, USA) (16) using “network (14)” and “network graph” packages (17).

### Inconsistency

Inconsistency evaluation was done using both global and local approaches. Loops specific approach was also used to detect loops of evidence for inconsistency (18, 19).

Quality or certainty of evidence was determined by using methodology as described by Salanti et al. (20) using Confidence in Network Meta-analysis (CINeMA) web application (21) for the primary outcome. CINeMA requires data formatted in terms of study level outcome, risk of bias and indirectness. Data was then configured and network plot was created. Nodes were colored green, yellow or red according to risk of bias (low, unclear and high, respectively). Edges of plot were colored according to average risk of bias across all the studies. Edging with was according to sample size and node size by number of studies. Then random-effect analysis with risk ratio as effect measure was used. A bar graph depicting contributions of each study to network estimate was generated. For this given network estimate risk of bias across contributions was summarized by selecting “Average” command. For assessing imprecision, a risk ratio of 1.25 was set as clinically important size of effect. Relative effect estimates below 0.8 and above 1.250 were considered clinically important. Judgment for imprecision was formulated as “very serious”, “serious” and “not serious” depending upon whether the confidence interval (CI) values cross both, one or neither limits of clinically important effect zones. Prediction intervals were generated to make judgments on heterogeneity and its implications on quality of treatment effects.

Incoherence or inconsistency was assessed according to methods described on separate section. Finally, results of all comparisons were graded as high, moderate, low or very low according to this framework.

## RESULTS

### Literature search and study characteristics

**Figure 1:**
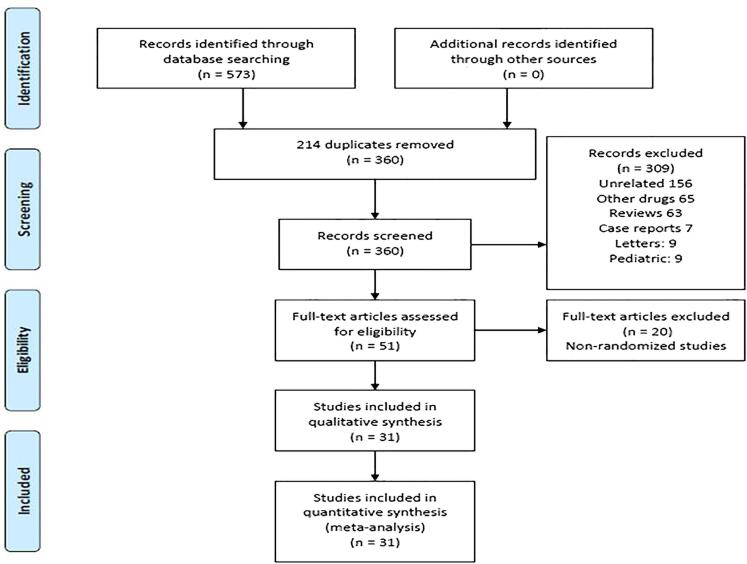
PRISMA flow-chart depicting search strategy used for conducting this study.

The literature search of various databases yielded a total of 573 citations that were imported on a citation manager. Of these 214 duplicate articles were removed and another 309 articles were removed after initial title and abstract screening due to various reasons (Figure-1). Fifty articles were selected for full-text review. For the final analysis 31 articles were included and the remaining 19 articles were excluded due to non-randomized nature of the study.

In this review, 31 RCT’s (12, 22-51) across various countries with 7077 patients were included.

The intervention group included the three alpha-blockers with or without best medical therapy i.e. adequate hydration and analgesic use. The control group was variable with 14 studies containing placebo groups and others containing best medical therapy and analgesics. Duration of treatment ranges from 10 days to 6 weeks. The duration of treatment was 4 weeks in 21 studies, 3 weeks in one study and 2 weeks in 3 studies. All the studies compared well for various demographic factors such as age and sex. The mean stone size was similar in the two groups in all the studies (Table-1).

**Table 1:** Characteristics of the studies included in this review.

S. no	Author / Country	Treatment	Control	Duration of treatment	Age mean (SD) / (Treatment) (Years)	Age mean (SD) (Control) / (Years)	Male/ Female (Treatment)	Male/ Female (Control)	Stone size less than 10 mm	Mean stone size (Treatment) (mm)	Mean stone size (Control) (mm)
1	Al-Ansari et al., (22), 2010, Qatar	Tamsulosin 0.4mg	Placebo	4 weeks	37.1(9.4)	36.1 (9.3)	32/18	35/15	Yes	5.8 (2.4)	6 (2.5)
2	Aldemir et al., (23), 2011, Turkey	Group I- Tamsulosin 0.4mg	Diclofenac 100 mg	10 days	42.4 (16)	43.5 (16.6)	22/9	19/10	Yes	6.7 (1.4)	6.6 (1.7)
		Group II- Rowatinex 100 mg thrice daily			46.5 (16.5)		17/13			6.8 (2)	
3	Alizadeh et al., (24), 2014, Iran	Tamsulosin 0.4 mg	Placebo	4 weeks	-	-	29/21	32/14	3-6 mm	-	-
4	Bajwa et al., (25), 2013, Pakistan	Tamsulosin 0.4 mg	Diclofenac 50 mg	4 weeks	32.4 (8.3)	33.8 (9.6)	18/12	19/11	Yes	6.9 (1.4)	6.6 (1.4)
5	Cervenakov et al., (26), 2002, Slovakia	Tamsulosin 0.4 mg	Tramadol 50 mg / Diazepan and Veral 50	-	-		32/19	33/18	Yes	-	-
6	El-Gamal et al., (27), 2011, Egypt	Group II Tamsulosin 0.4mg	Group I Placebo control Group III UralytU	4 weeks	35.3(5.7)	36.2 (6)	32/16	34/12	Yes	7.9 (1.9)	7.7 (1.6)
		Group IV Uralyt-U plus tamsulosin									
7	ElGalaly et al., (28), 2017, Egypt	Group I Tamsulosin 0.4 mg	-	4 weeks	35.5 (11)	-	32/19	-	Yes	5.6( 1.2)	-
		Group II Silodosin 8mg			33.6 (9.9)		35/17			5.4(1.5)	
8	Vincendeau et al., (29), 2010, France	Tamsulosin 0.4 mg	Placebo	6 weeks	38.9 (12)	39 (11)	43/18	52/9	2-7 mm	2.9 (1)	3.2 (1.2)
9	Yilmaz et al., (30), 2005, Turkey	Group II Tamsulosin 0.4mg Group III	Group I No treatment	4 weeks	40.6(10)	41.6(12)	9/20	19/9	Yes	6(1.2)	6(1.4)
		Terazosin 5mg Group IV Doxazosin 4mg									
10	Aggarwal et al., (31), 2009, India	I – Tamsulosin	III -Placebo	4 weeks	(31.4)	(35.3)	26/8	24/10	Yes	6.17	6.35
		II – Alfuzosin			(38.7)		28/6			6.7	
11	Ahmad et al., (32), 2015, Pakistan	I -Tamsulosin	II- Placebo	4 weeks	-	-	4-10mm		<8mm	5.78mm	-
12	Cha et al., (33), 2012, Korea	ITamsulosin - 0.2mg OD	-	4 weeks	(45.07)	-	31/10	-	4-10mm	5.49	-
		II Tamsulosin - 0.2 mg BD									
		III Alfuzosin									
		IV Trospium									
13	Dell’Atti et al., (34), 2015, Italy	I Tamsulosin	-	3 weeks	(35)	-	39/27		4-10mm	5.37	-
		II - Silodosin									
14	Furyk et al., (35), 2016, Australia	I -Tamsulosin	II-Placebo	4 weeks	> 18	>18	156/42	164/31	Yes	4	3.7
15	Ochoa-Gomez et al., (36), 2011, Mexico	I- Tamsulosin	II-Placebo	4 weeks	(38.5)	(38.2)	15/17	21/12	5-10mm	5.3	5.2
16	Hermanns et al., (37), 2009, Switzerland	I-Tamsulosin	II-Placebo	3 weeks	(36)	(41)	39/6	36/9	7mm or less	4.1	3.8
17	Itoh et al., (38), 2013, Japan	II- Silodosin	I -Placebo	4 weeks	(56.3)	(55.8)	All male	-	Yes	4.87	5.07
18	Kumar et al., (12), 2015, India	I - Tamsulosin	-	4 weeks	(36.4)	-	62/28	-	5-10mm	7.44	-
		II - Silodosin									
		III - Tadalafil									
19	Sameer et al., (39), 2014, India	I-Nifedipine II- Alfuzosin	III- Control	4 weeks	(32.74)	(33.06)	19/16	23/12	Yes	6.5	6.37
20	Ahmad et al., (40), 2010, Saudi Arabia	I = Tamsulosin 0.4mg	III- control- Diclofenac 75mg	30 days	40.7(14.8)	38.9(13.3)	9/10	19/09	Yes	4.97 (2.24)	5.39 (1.81)
		II - Alfuzosin 10mg									
21	Elsaid et al., (41), 2015, Egypt	Alfuzosin 5mg BD	control - Diclofenac + Hydration	4 weeks	32.8(9.5)	32.1(9.2)	18/10	16/10	Yes	6.3 (2.1)	5.9 (1.9)
22	Nuraj et al., (42), 2017, Kosovo	I-Tamsulosin 0.4mg	Control- Diclofenac	4 weeks	35.5(11.0)	35.4(10.8)	34/18	35/17	4-10mm	6.5 (1.6)	6.6 (1.5)
23	Pedro et al., (43), 2008, USA	I- Alfuzosin	Placebo	4 weeks	36.69(13.6)	42.03(12.8)	28/6	27/8	Up to 8mm	3.83 (0.94)	4.07 (1.13)
24	Pickard et al., (44), 2015, UK	I - Tamsulosin 0.4mg	III- Placebo	4 weeks	43.1(11.5)	48.2(12.3)	315/68	299/85	Yes	4.6(1.6)	4.5(1.7)
		III - Nifedipine 30 mg									
25	Rahman et al., (45), 2018, India	I - Tamsulosin 0.4mg OD	-	4 weeks	38(10)	-	24/16	-	5-10mm	7.5(1.20)	
		II - Silodosin 8 mg OD									
		III- Silodosin 8mg + Tadalafil 5 mg									
26	Sur et al., (46), 2015, USA	I- Silodosin 8mg OD	II- Placebo	4 weeks	47 (13)	47 (15)	72/53	80/37	4-10mm	5.4 (1.4)	5.5 (1.6)
27	Wang et al., (47), 2008, Taiwan	I- Tamsulosin 0.4 mg OD	III- control	2 weeks	50.4(9.7)	50.9(9.6)	22/10	23/08	Yes	6.5(1.3)	6.5(1.4)
		II- Terazosin 2 mg OD									
28	Ye et al., (49), 2018, Wuhan, China	I- Tamsulosin 0.4mg	II- Placebo	4 weeks	40.1(11.6)	40.7(12.3)	556/1086	605/1049	Yes	5.8(1.9)	5.7(1.8)
29	De Sio et al., (50), 2006, Italy	Diclofenac 100mg/day + Aescin 80mg/day	-	2 weeks	44.5(11.3)	-	26/20	-	Yes	6.4(1.3)	-
		Diclofenac 100mg + Aescin 80mg + Tamsulosin 0.4 mg									
30	Wang et al., (48), 2016, Taiwan	I- Silodosin 8mg	II- control	2 weeks	51.4(8.6)	51.5(10.5)	40/22	43/18	Yes	6.4(1.4)	6.4(1.3)
31	Yuksel et al., (51), 2015, Turkey	Group II Silodosin 4 mg/day	Group I Placebo	3 weeks	35.31 (11.55)	35.23 (11.2)	19/16	20/15	4-10mm	6.40(1.61)	6.34(1.57)

## NETWORK

Data for primary outcome i.e. SER was available from all the 31 studies. Mixed evidence was available for 5 comparisons (alfuzosin vs. control, silodosin vs. control, tamsulosin vs. control, alfuzosin vs. tamsulosin and silodosin vs. tamsulosin) whereas indirect evidence was available for alfuzosin vs. silodosin. The network plot for the primary outcome is shown in APPENDIX 1. As described previously, nodes were colored green, yellow or red according to risk of bias (low, unclear and high, respectively). Edges of the plot were colored according to the average risk of bias across all the studies. Edge width was according to the sample size and node size by the number of studies. The number of studies for each comparison are alfuzosin vs. control (5), silodosin vs. control (4), tamsulosin vs. control (19), alfuzosin vs. tamsulosin (3) and silodosin vs. tamsulosin (4). For other outcome i.e. SET network consists of 19 studies with 4 treatments and 5 comparisons (alfuzosin vs. control (3), silodosin vs. control (2), tamsulosin vs. control (11), alfuzosin vs. tamsulosin (2) and silodosin vs. tamsulosin (3).

### Stone expulsion rate

Compared to placebo all the treatment groups were more effective. Relative risk (RR) of stone expulsion rate for silodosin, alfuzosin and tamsulosin were 1.55 (95% confidence interval (CI) 1.31, 1.83), 1.33 (95% CI 1.06, 1.67) and 1.29 (95% CI 1.16, 1.43) respectively. Compared to alfuzosin RR of silodosin and tamsulosin were 1.16 (95% CI 0.9, 1.51) and 0.97 (95% CI 0.78, 1.19) respectively.

**Figure 2:**
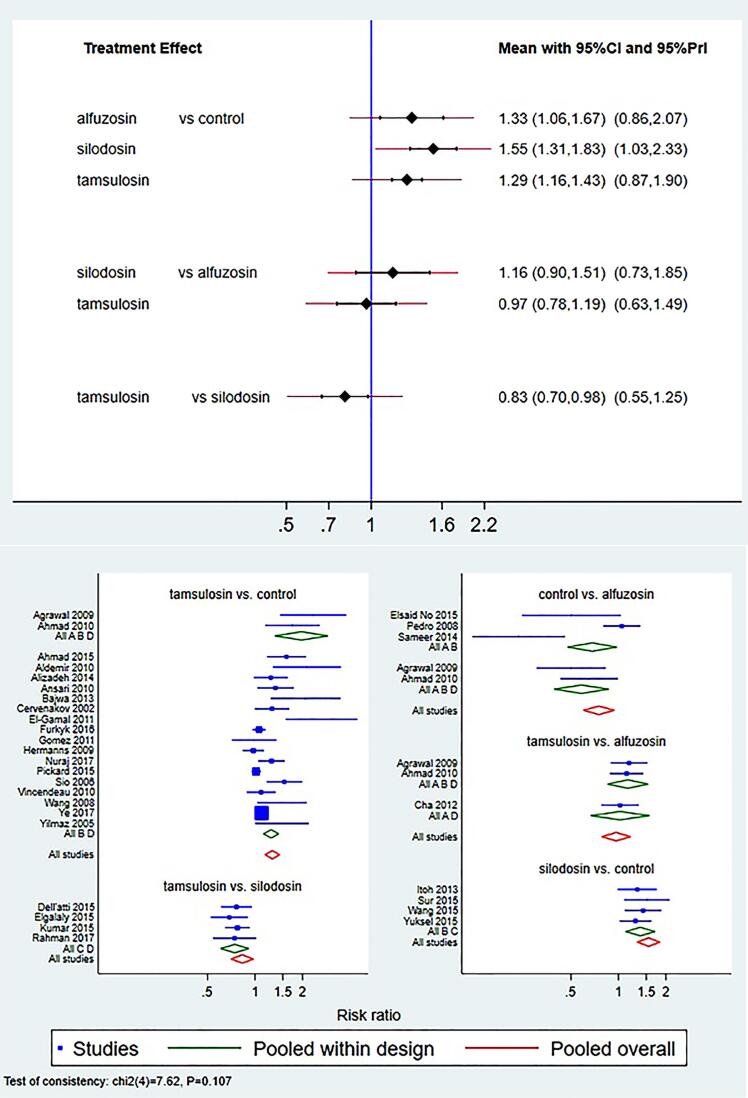
Interval and Forest plot for various drugs for stone expulsion rate (SER).a

Comparison of tamsulosin with silodosin had RR of 0.83 (95% CI 0.70, 0.98) (Figure-2).

Global approaches for inconsistency models revealed no violation of consistency assumption for direct and indirect assumptions. Loop specific approach revealed two treatment loops without inconsistency. Local approaches using node splitting revealed inconsistency for two comparisons i.e. alfuzosin-silodosin and alfuzosin-tamsulosin. SUCRA values were calculated to estimate the rank of efficacy according to stone expulsion rate (Table-2). According to the SUCRA values obtained silodosin had the highest rank followed by alfuzosin and tamsulosin.

**Table 2:** Surface under cumulative ranking area (SUCRA) values according to various outcomes.

TREATMENT	SUCRA for SER	SUCRA for SET	SUCRA for Postural hypotension	SUCRA for retrograde ejaculation
**Control**	0.1	0.2	35.7	32.1
**Alfuzosin**	58.8	64.9	**86.1**	6.8
**Silodosin**	**94.8**	**90.4**	17.6	**98.6**
**Tamsulosin**	46.2	44.5	60.6	62.6

**SER** = Stone expulsion rate; **SET** = Stone expulsion time

### Time to stone expulsion

**Figure 3:**
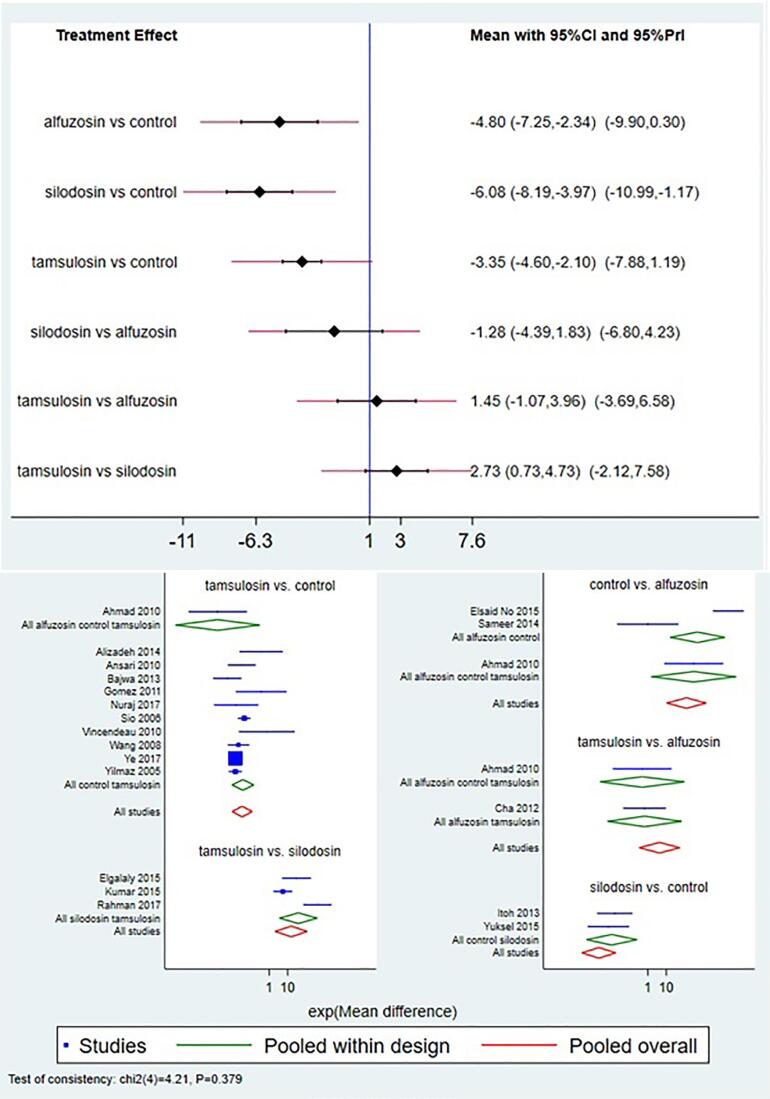
Interval and Forest plot for various drugs for stone expulsion time (SET).

Data on the time to stone expulsion was available from 18 studies. Comparison of silodosin to other treatment modalities such as control, alfuzosin and tamsulosin favored silodosin with mean difference (MD) of -6.0 (95% CI, -8.1, -3.9) `days, -1.28 (95% CI -4.4, -1.8) days, 2.73 (95% CI 0.73, days respectively. Comparison of tamsulosin with control favored tamsulosin with MD of -3.35 (95% CI -4.6, -2.1) and comparison to alfuzosin favored alfuzosin with MD of 1.45 (95% CI -1.07, 3.96) Comparison of alfuzosin with control group favored alfuzosin with MD of -4.8 (95% CI -7.25, -2.3) (Figure-3).

Loop-specific heterogeneity estimates and inconsistency models (global and local) revealed no violation of consistency assumption for direct and indirect assumptions SUCRA values obtained for time to stone expulsion estimate revealed highest values for silodosin i.e. silodosin was best with lowest SET followed by alfuzosin and tamsulosin (Table-2).

### Complications

**Figure 4:**
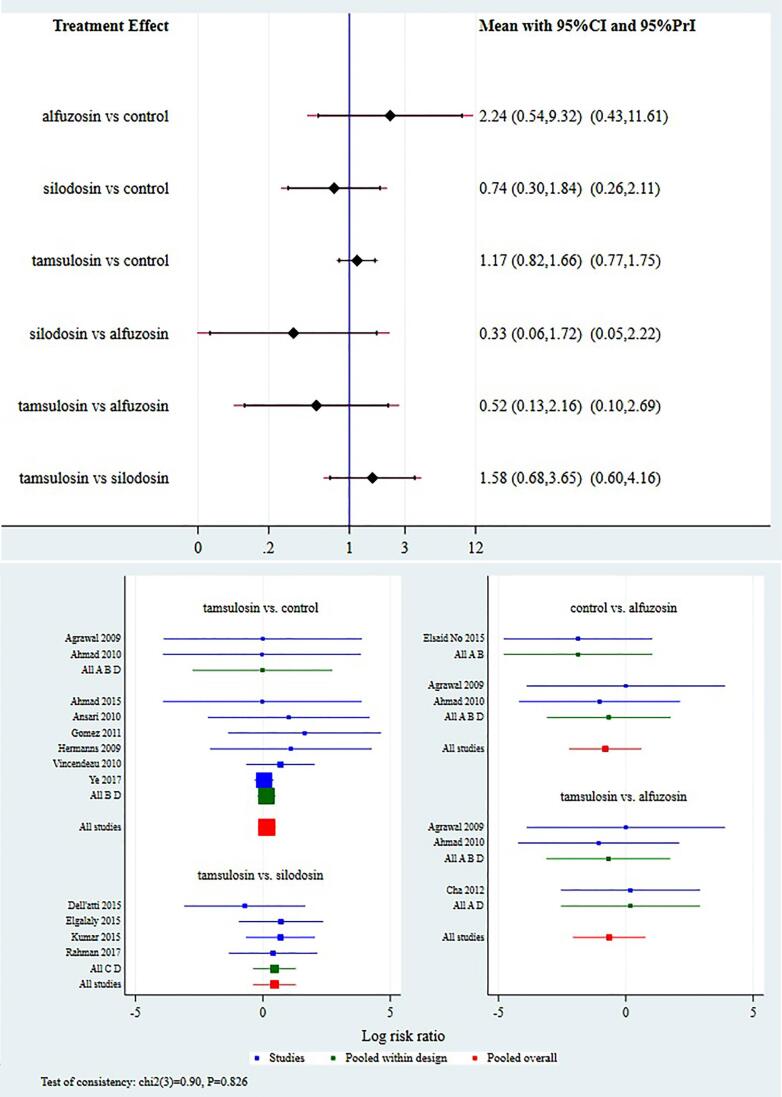
Interval and Forest plot for various drugs for postural hypotension.

**Figure 5:**
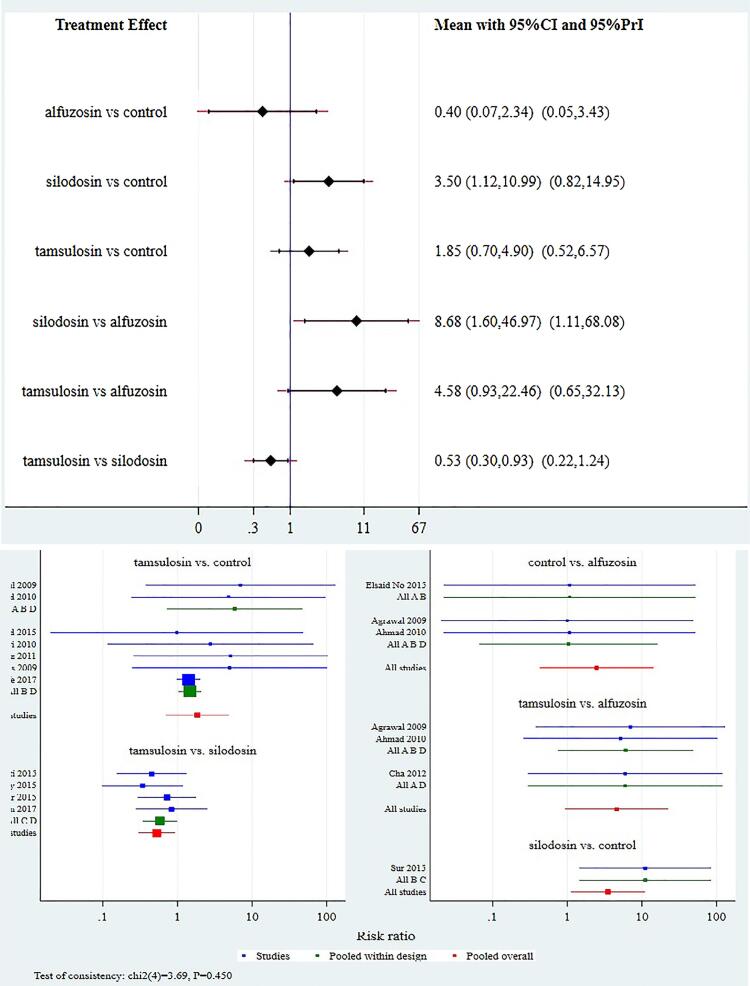
Interval and Forest plot for various drugs for retrograde ejaculation.

Data for analysis into network meta-analysis has been inconsistently reported. For this study, we extracted data for two commonly reported and clinically relevant side-effects i.e. postural hypotension and retrograde ejaculation. Data for postural hypotension and retrograde ejaculation was available from 14 studies. For postural hypotension there was no significant difference between all the treatment and control groups (Figure-4). According to SUCRA values alfuzosin had the highest ranked and silodosin the lowest ranked i.e. silodosin had lowest incidence of postural hypotension (Table-2). For retrograde ejaculation, silodosin had significantly higher incidence as compared to all the other treatment groups (Figure-5). According to SUCRA values, silodosin had the highest incidence of retrograde ejaculationand alfuzosin the least (Table-2).

### Sensitivity analysis

Sensitivity analysis was performed according to risk of bias and duration of treatment. Analysis after excluding studies at high risk bias was performed and we found RR of 1.48, 1.12, 1.18 for silodosin, tamsulosin and alfuzosin respectively as compared to placebo. Again, silodosin was the highest ranked drug followed by alfuzosin.

Separate analysis for studies with treatment duration for 4 weeks was also performed including 21 studies. SUCRA values obtained again revealed similar results. Similar results were obtained when analysis of studies with 4 weeks of follow-up at low or unclear risk of bias was performed (excluding studies at high risk of bias) Visual interpretation of comparison adjusted funnel plots revealed mild asymmetry for both the outcomes (Supplementary APPENDIX 2).

### Certainty of evidence

Study level risk of bias assessment as per Cochrane risk of bias tool revealed 13 studies at low risk bias, 4 at unclear risk and 14 at high risk of bias. Mixed evidence for all the comparisons was obtained except for alfuzosin-tamsulosin for which only indirect evidence was available. Overall assessment of results of confidence in certainty of evidence for each comparison group is presented in the supplementary file S3.

## DISCUSSION

For the last few decades, MET has been routinely used for conservative management of ureteric stones owing to its increased expulsion rate, accelerated “time to expulsion”, and decreased severity of colic episodes (7). Studies in the past have shown that various types of pharmacotherapy like alpha- blockers, CCB, PDE-5 inhibitors, and steroids have better efficacy than placebo in management of the ureteric calculi (52). Few meta-analyses have also examined the role of various combinations of drug interventions in the management of ureteric stones (53), however, there is a paucity of studies specifically comparing the different alpha-blockers for treatment of distal ureteric calculi. Moreover, due to lack of studies comparing “head-to-head” interventions the most appropriate choice to treat ureteric stones remains unknown. This drawback can be circumvented by a “Network meta-analysis”, in which both direct and indirect comparisons are analyzed with a common control within a network framework. With NMA approach researchers could acquire direct and indirect evidence through the comparison with a common comparator, thus making it possible to assess the actual effectiveness ranking of numerous interventions (54). Therefore, we did the present network meta-analysis to evaluate the relative efficacy of the commonly used alpha-blockers in the management of distal ureteric calculi.

### Quality of the evidence and implications for research

One important concern in this review is that there were 14 studies at overall high risk of bias due to inadequate data in the allocation concealment and the detection bias domains. Overall confidence for each comparison group in this review ranged from “very low” to “moderate” according to CINeMA approach. Same care was taken during our sensitivity analysis by excluding studies at a high risk of bias and we still found our results to hold true.

In this large network meta-analysis, 31 randomized controlled trials enrolling 7077 patients with lower ureteric stones less than 10mm were incorporated in our final analysis. We compared SER and SET to assess the comparative efficacy of the commonly used alpha-blockers (alfusozin, silodosin and tamsulosin) for the treatment of distal ureteric stones. Previously, numerous meta-analyses have been published evaluating different alpha-blockers like tamsulosin (55), silodosin (56), and alfuzosin (57) and all have reported that the studied alpha-blockers are more efficacious than the control or placebo in improving stone clearance and time to stone expulsion. However, few multi-centre RCTs have demonstrated no significant favourable efficacy of alpha-blockers on the clearance rates as compared to placebo (35, 44, 46). But, majority of these trials (35, 44), have nearly two-thirds of the patient with stones smaller than 5mm and since smaller stones have more likelihood of spontaneous expulsion, even in the absence of MET, the potential favourable effect of MET for smaller stones seems non-significant. Our study has concluded that alpha-blockers are definitely superior to placebo or control in terms of better SER and lesser time to stone expulsion.

Regarding the differences amongst the individual alpha-blockers, a meta-analysis completed by Sridharan et al. (52), concluded that silodosin was superior to others for SET and terazosin has the highest SER. While, a systematic review published by Campschroer et al. (58) concluded that the efficacy of alpha-blockers does not depend on the type of alpha-blocker used. Therefore, the results of this meta- analysis are different from the previous meta-analysis and silodosin appears to be the most efficacious drug in terms of SER and SET. From the side effect profile, all the drugs in this meta-analysis had similar incidence of postural hypotension, whereas silodosin had significantly higher incidence of retrograde ejaculation. Thus, silodosin may be the most efficacious drug in terms of SET and SER for the distal ureter stones; however, it may not be suitable for patients who are concerned about sexual function.

Alfuzosin seems to be suitable alternative for such patients.

### Limitations

The present study is not without limitations. Firstly, we did not perform a subgroup analysis based on stone size as data was lacking from most of the studies. Secondly, many studies in the present analysis were not placebo-controlled and were at high risk of bias. Thirdly, apart from postural hypotension and retrograde ejaculation other adverse events were not explicitly studied in the included studies. Lastly, the literature search was limited to English language only.

## CONCLUSIONS

Our NMA suggested that silodosin has highest SER followed by alfuzosin and tamsulosin. The time to stone expulsion is lower with silodosin followed by alfuzosin and tamsulosin. Therefore, silodosin appears to be the most efficacious drug of the three for lower ureteric stones less than 10mm.

## ABBREVIATIONS

CCB: Calcium channel blockers
CINeMA: Confidence in Network Meta-analysis
NMA: Network meta-analysis
MET: Medical expulsive therapy
PDEI: Phosphodiesterase inhibitors
SER: Stone expulsion rate
SET: Stone expulsion time
SUCRA: Surface under cumulative ranking curve

## APPENDIX 1

[Fig f6]

## APPENDIX 2

[Fig f7]
